# Maternal malaria status and metabolic profiles in pregnancy and in cord blood: relationships with birth size in Nigerian infants

**DOI:** 10.1186/1475-2875-11-75

**Published:** 2012-03-19

**Authors:** Omolola O Ayoola, Andrew Whatmore, Williams O Balogun, Olatokunbo O Jarrett, John K Cruickshank, Peter E Clayton

**Affiliations:** 1 Manchester Academic Health Sciences Centre, University of Manchester, Paediatric Endocrinology, 5th Floor (Research), Royal Manchester Children's Hospital, Oxford Road, Manchester M13 9WL, UK; 2 College of Medicine, University of Ibadan, Ibadan, Nigeria

## Abstract

**Background:**

Malaria is more common in pregnant than in non-pregnant Nigerian women, and is associated with small birth size and the attendant short- and long-term health risks. The influence of malaria on maternal metabolic status in pregnancy and in cord blood and how this relates to birth size has not been studied. The study objective was to define relationships between maternal and cord serum metabolic markers, maternal malaria status and birth size.

**Methods:**

During pregnancy, anthropometric measurements, blood film for malaria parasites and assays for lipids, glucose, insulin and TNF were obtained from 467 mothers and these analytes and insulin-like growth factor-I (IGF-I) were obtained from cord blood of 187 babies.

**Results:**

Overall prevalence of maternal malaria was 52%, associated with younger age, anaemia and smaller infant birth size. Mothers with malaria had significantly lower cholesterol (total, HDL and LDL) and higher TNF, but no difference in triglyceride. In contrast, there was no effect of maternal malaria on cord blood lipids, but the median (range) cord IGF-I was significantly lower in babies whose mothers had malaria: 60.4 (24,145)μg/L, *versus *no malaria: 76.5 (24, 150)μg/L, *p *= 0.03. On regression analysis, the key determinants of birth weight included maternal total cholesterol, malarial status and cord insulin and IGF-I.

**Conclusions:**

Malaria in pregnancy was common and associated with reduced birth size, lower maternal lipids and higher TNF. In the setting of endemic malaria, maternal total cholesterol during pregnancy and cord blood insulin and IGF-I levels are potential biomarkers of foetal growth and birth size.

## Background

In Nigeria, increasing mortality from stroke and end-stage renal failure is associated with a high prevalence of hypertension, obesity, diabetes and high serum triglycerides especially in women [1]. Numerous studies have established associations between reduced birth weight and increased risk of coronary heart disease, diabetes, hypertension and stroke in adulthood [2,3]. Malaria remains endemic in Nigeria and is more common among pregnant women, with prevalence ranging from 20% to 44%. It leads to significant consequences for maternal and infant health, such as maternal anemia, responsible for 11% of maternal deaths, and low birth weight (LBW), responsible for 5-12% of all LBW, 43% of preventable LBW babies and contributes to 75,000-200,000 infant deaths each year in Nigeria [4-9]. Therefore, malaria in pregnancy may contribute to later life morbidities in keeping with the 'developmental origins' hypothesis.

The biologic mechanisms underlying this hypothesis are poorly understood and mediators of the relationships between LBW, malaria in pregnancy and later cardiovascular and metabolic morbidity have not been clearly identified. Few studies have examined the relationships between maternal metabolic markers, foetal hormones and birth weight, nor has the effect of malaria in pregnancy on these relationships been explored. Maternal and cord blood levels of lipids, glucose and insulin and cord insulin-like growth factor-I (IGF-I) levels have been investigated as possible determinants of birth weight [10-12]. Maternal fasting triglyceride (TG) was independently associated with birth weight in non-diabetic women with maternal hyperglycaemia and with foetal growth and birth size in women with gestational diabetes [11,13,14]. Maternal plasma glucose was positively associated with birth weight in non-diabetic women [10,15]. The relationship between birth weight and cord blood lipids was inconsistent [16,17], but positive correlations between birth weight and cord blood glucose and insulin levels in both normal and low birth weight babies have been reported [18]. Cord blood IGF-I may also be involved in the control of foetal size during late gestation [19,20]. Increased placental expression of cytokines such as tumour necrosis factor (TNF), interleukin 8, γ-interferon, IL-6 and IL-10 occurred in pregnancies affected by malaria, but only TNF has been linked to LBW [21,22].

This study sought to explore the hypothesis that malaria parasitaemia in pregnancy would induce changes in maternal metabolic markers, which would be associated with reduced birth size. The relationships between maternal and cord blood metabolic profiles and birth size in the setting of endemic malaria was examined and potential pregnancy biomarkers of LBW were identified using an established cohort of mothers and infants born in Nigeria. [23]

## Methods

### Study site

The study was carried out in Yemetu-Adeoyo, a semi-urban community in Ibadan in south-west Nigeria where transmission of malaria is perennial. The hospital in this community, Adeoyo Maternity Hospital (AMH), is the oldest maternity hospital in Nigeria, dating from 1927. There are over 4,000 deliveries annually. Ethical approval for the study was obtained from the joint University of Ibadan/University College Hospital ethical committee and the University of Manchester Ethics committee.

### Study procedures, follow-up, delivery and recruitment of babies

Healthy pregnant women aged 18-45 years presenting at AMH before 36 weeks' gestation and all babies born ≥ 37 weeks' gestation were eligible. Women who were HIV positive or had sexually transmitted infections at booking, those with pre-term deliveries, as well as those with multiple pregnancies or with chronic diseases, such as hypertension and diabetes were excluded. Babies with known syndromes, metabolic defects, major congenital abnormalities or severe birth trauma were also excluded.

Standard operating procedures (SOP) were developed and women were enrolled over one year to cover both wet and dry seasons. The study protocol and the rationale for the study were explained carefully in appropriate language, most commonly Yoruba or English, with questions answered as needed and written informed consent was obtained from all participants. After the delivery of their babies, another written informed consent was also obtained for the participation of the babies in the study.

Information on socio-demographic, obstetric, family, and health history including malarial frequency and use of anti-malarial drugs, was collected. All participants were issued prescriptions of sulphadoxine-pyrimethamine for intermittent preventive therapy (IPT) for malaria according to standard hospital practice.

Antenatal visits followed routine practice with frequency of attendance determined by gestational age. Standardized measures of anthropometry were carried out on all women at every visit until delivery. Maternal weight was measured to the nearest 0.1 kg on a SECA scale, and height on a stadiometer, both without shoes according to the SOP.

### Visit schedule and blood measurements

At booking, 2 ml of blood was collected into an EDTA tube for full blood count and blood films for malaria parasites (MP) were prepared, stained with 3% Giemsa at pH 7.2. Repeat thick blood films for MP were obtained at every subsequent visit and at delivery. In addition, the placenta was weighed, turned to the maternal surface, cotyledons exposed and 1 ml of blood obtained from the inter-villous space for a placental malarial blood smear.

Blood films were examined for MP under light microscopy and recorded as negative only after 100 or more high-power microscope fields had been scanned. In those with malaria, parasite densities were determined by counting the number of parasites (np) among 200 leucocytes on the thick film using the following equation: Absolute parasite counts = (np x TLC)/200 where TLC = subject's total leucocyte count. For quality control, 30% of negative samples and 40% of positive samples were re-examined by two different, trained microscopists.

At the second antenatal visit, of 624 healthy pregnant women enrolled, 467 women gave their consent for fasting blood to be taken for biochemical markers while 396 of them had samples for malaria parasites. At delivery, 27 were excluded due to four maternal deaths (0.9%), 11 still births (2.4%), five miscarriages (1.1%) and seven neonatal deaths (1.5%), leaving 436 mother-baby pairs.

Cord blood was collected from the umbilical vein on the foetal surface of the placenta. Plasma was separated by centrifugation at 3,000 rpm and 4°C for 10 minutes and aliquoted into microtubes and stored at -80°C prior to lipid, glucose, insulin, TNF and IGF-I (cord blood only) assays. Cord glucose could not be measured immediately and so was not included in the assays.

Based on availability of complete maternal and cord blood samples, 187 mother-baby pairs were selected. There were no significant differences in the socio-demographic and clinical data of excluded women.

### Definitions

Malaria was defined as the presence of asexual blood stages of *Plasmodium falciparum *in peripheral blood. There were two definitions of malaria:

a) 'Malaria at recruitment' = Malaria at second antenatal visit when blood for biochemical markers was also obtained. This was only used in analyses of effects on maternal biochemical markers at the same time-point. There were 72 blood smears positive for malaria parasites out of the 396 samples giving the prevalence of malaria as 18%.

b) 'Maternal malaria' = Malaria parasitaemia present at least once during pregnancy and/or at delivery (n = 211). These included (i) malaria parasitaemia in peripheral blood at least once during pregnancy (n = 138); and (ii) at delivery and/or in the placenta (n = 73). This was used in analyses of relationships with cord parameters and effects on birth indices.

Out of 187 mother-baby pairs with complete maternal and cord blood samples, 97 had malaria giving a prevalence of 52%.

Anaemia was defined as a packed cell volume (PCV) < 30%.

### Biochemical assays

Total cholesterol (TC), High-density lipoprotein-cholesterol (HDL-C) and triglyceride concentrations were determined by using standard enzymatic procedures on an automatic analyser (COBAS MIRA/HITACHI 704 - Roche Diagnostics, Germany). The inter- and intra-assay coefficients of variation (CVs) for all parameters were < 5%. Low-density lipoprotein- cholesterol (LDL-C) was calculated using the Friedewald formula [24].

The normal values of lipids (mmol/L) in adult women are TC 3-5, HDL-C 1.2-2.2, LDL-C 2-3 and TG 0.6-1.68.

Maternal glucose was measured by the glucose oxidase method using a commercial kit (Randox, Crumlin, UK) on a YSI 2300 stat plus analyser (YSI, Farnborough, Hants, UK). The intra-assay CV was 1.5% at 4.1 mmol/L, and inter-assay CVs were 2.8% and 1.7% at 4.1 and 14.1 mmol/L respectively. Normal fasting glucose values are 3.9-6 mmol/L.

Insulin was measured by ELISA, using a commercial kit (Mercodia, Uppsala, Sweden). Assay sensitivity was 1 mU/L. Intra-assay CVs were 3.4% and 3.2% at 11 and 154 mU/L, and equivalent inter-assay CVs were 3.6% and 2.9%.

IGF-I and TNF were measured using Immulite 2000 assays (DPC, Lumigen Inc, Southfield, UK). Respective assay sensitivities were 25 μg/L and < 0.09 ng/L. Inter-assay CV values for IGF-1 at 48.9 and 158.5 μg/L were 7.6 and 9.2%. For TNF, intra-assay CVs were 6.7% and 5.3% at 6.3 and 19 ng/L, and the inter-assay CVs at 6.1 and 18.6 ng/L were 8.2 and 9.7% respectively.

### Infant anthropometry

Babies were measured within 72 hours of birth. They were weighed naked to the nearest 0.1 kg and length measured on an infant stadiometer from crown to heel to the nearest 0.1 cm. Occipito-frontal circumference (OFC) was taken around the widest circumference of the head using a non-stretchable tape. Skinfold thicknesses (triceps, biceps, sub-scapular, and suprailiac) were measured using Holtain calipers on the left side to the nearest 0.1 mm. Measurements were obtained in duplicate or triplicate if disagreeing by > 15%.

### Validity of anthropometric measurements

Based on the WHO Manual (1995), three nurses, already proficient in paediatric venipuncture, were trained in anthropometry methods. They carried out all measurements on the same equipment throughout the study. They also had three-monthly protocol-refresher training sessions and used training videos to minimize inter-observer and within-observer errors.

### Statistical analysis

Data were analysed using SPSS version 14 (SPSS Inc, Chicago, IL). Results were expressed as mean (SD) or median (IQ range), using Student t/Mann Whitney and Chi square tests for associations between maternal and infant clinical characteristics and malaria. Levels of insulin, IGF-I and TNF were skewed and, therefore, tested non-parametrically, while lipids and glucose levels were normally distributed and tested parametrically. Correlations were assessed by Spearman's test. All tests were two-sided and p values < 0.05 were considered significant.

Simple linear regression was used to assess the determinants of infant size, indicating those with *p *< 0.001, *p* < 0.01 and < 0.05. Due to high co-linearity between biochemical parameters, a stepwise method of model selection was used to identify the key variables.

## Results

The age and BMI of mothers were similar irrespective of trimester at recruitment. Mean (SD) gestational ages at booking and at delivery were 27.1 (5.1) and 39.3 (1.4) weeks respectively (Table 1).

**Table 1:** Maternal clinical characteristics and biochemical markers at recruitment

Parameter (n = 467)	Trimester 2 n = 125	Trimester 3 n = 342	P value^a, b^
**Age **(year)	29.0 (4.9)	28.5 (5.3)	0.280

**Gestational age **(weeks)	22.8 (3.2)	31.9 (3.3)	** < 0.001**

**BMI **(kg/m^2^)	25.2 (3.9)	24.9 (3.9)	0.501

**Total cholesterol **(mmol/L)	4.44 (3.93, 5.45)	4.98 ((4.28, 5.69)	**0.002**

**HDL-C **(mmol/L)	1.54 (1.27, 1.83)	1.55 (1.27, 1.81)	0.922

**LDL-C **(mmol/L)	2.49 (1.97, 3.13)	2.74 (2.17, 3.25)	**0.021**

**Triglyceride **(mmol/L)	1.31 (1.05, 1.51)	1.47 (1.23, 1.79)	** < 0.001**

**Glucose **(mmol/L)	4.33 (4.04, 4.55)	4.13 (3.87, 4.41)	** < 0.001**

**Insulin **(mU/L)	4.32 (2.76, 6.90)	3.92 (2.21, 6.09)	0.204

**TNF **(ng/L)	0.17 (0.09, 0.83)	0.35 (0.09, 0.83)	0.102

**Insulin **(mU/L)	4.32 (2.76, 6.90)	3.92 (2.21, 6.09)	0.204

**TNF **(ng/L)	0.17 (0.09, 0.83)	0.35 (0.09, 0.83)	0.102

Variables expressed as either Mean (SD) or Median (IQ Range)

^a ^Student t test done for variables presented as mean (SD);

^b^Mann Whitney U test for variables presented as median (IQ range)

BMI, Body mass index; HDL-C, High-density lipoprotein-cholesterol; LDL-C, Low-density lipoprotein-cholesterol; TNF-α, Tumour necrosis factor-alpha

Plasma levels of all lipids except HDL-C were significantly elevated in the third compared to the second trimester but fasting plasma glucose was reduced. Insulin and TNF levels were similar.

### Effect of maternal malaria at recruitment on maternal characteristics and biochemical markers

The prevalence of malaria among the women at recruitment was 18%. Among all women recruited, 28% were primigravid, and this was associated with MP (28% *vs *13% in the multigravid, *p *= 0.001). Primigravidity was associated with 2.5-fold increase in risk of having malaria [OR = 2.5, 95% CI, 1.5-4.2].

Most women were asymptomatic and only seven had fever. Malaria was also associated with younger maternal age (29.4 *vs *27.7 years, *p *= 0.001) and lower packed cell volume [31.7 (4.7) *vs *32.8 (3.3)%, *p *= 0.006].

Mothers with malaria had significantly lower TC, lower HDL-C, lower LDL-C, no change in TG but higher TNF in both the second and third trimesters (Table 2). There was no difference in glucose and insulin levels in the two trimesters with the presence of MP.

**Table 2:** Effect of malaria at recruitment on maternal biochemical markers

Maternal Biochemical Markers	Trimester 2 n = 110		P value	Trimester 3 n = 286		P value
			
Total n = 396	Malaria Absent n = 93	Malaria Present n = 17		Malaria Absent n = 231	Malaria Present n = 55	
**TC **(mmol/L)	4.73(4.26,5.71)	3.73(2.80,4.28)	**< 0.001**	5.17(4.50,5.81)	4.43(3.67,5.41)	**< 0.001**

**HDL-C **(mmol/L)	1.58(1.37,2.02)	0.86(0.70,1.38)	**< 0.001**	1.64(1.39,1.84)	1.27(0.97,1.63)	**< 0.001**

**LDL-C **(mmol/L)	2.66(2.03,3.28)	2.10(1.51,2.53)	**0.010**	2.77(2.31,3.43)	2.44(1.70,3.08)	**0.006**

**TG **(mmol/L)	1.29(1.02,1.51)	1.35(1.19,1.62)	0.112	1.47(1.24,1.77)	1.51(1.28,1.99)	0.125

**Glucose **(mmol/L)	4.31(4.06,4.58)	4.40(3.89,4.48)	0.795	4.10(3.85,4.37)	4.23(3.97,4.44)	0.060

**Insulin **(mU/L)	4.58(3.06,7.56)	2.95(1.87,5.10)	0.056	3.86(2.16,6.11)	4.34(2.66,6.06)	0.316

**TNF **(ng/L)	0.09(0.09,0.55)	0.79(0.45,1.91)	** < 0.001**	0.29(0.09,0.78)	0.55(0.09,1.76)	**0.003**

**Insulin **(mU/L)	4.58(3.06,7.56)	2.95(1.87,5.10)	0.056	3.86(2.16,6.11)	4.34(2.66,6.06)	0.316

**TNF **(ng/L)	0.09(0.09,0.55)	0.79(0.45,1.91)	** < 0.001**	0.29(0.09,0.78)	0.55(0.09,1.76)	**0.003**

Variables expressed as either Mean (SD) or Median (IQ Range)

TC, Total Cholesterol; HDL-C, High-density lipoprotein-cholesterol;

LDL-C, Low-density lipoprotein-cholesterol; TG, Triglyceride;

TNFα, Tumour necrosis factor-alpha

### Effect of maternal malaria on newborn birth indices and cord blood biochemical markers

Prevalence of maternal malaria defined as malaria in pregnancy and/or at delivery overall was 52%. Mean birth weight, length, OFC, mid-upper arm circumference (MUAC) and biceps skinfold thicknesses of infants born to women with malaria were globally smaller than those of women without malaria. Mean birth weight was significantly lower by 160 gms (-5.3% compared to babies of mothers without malaria), biceps skinfold thickness by 1.5 mm (-4.1%) and MUAC by 1.8 mm (-1.8%), while birth length and OFC were lower by only 6.2 cm (-1.3%) and by 3.5 mm (-1%) respectively (Table 3). The placental weights of infants born to women with and without malaria were not significantly different but the placenta-foetal weight ratios for infants whose mothers have malaria were significantly lower than those whose mothers did not have malaria (*p *= 0.019) (Table 3).

**Table 3:** Effect of maternal malaria on birth indices, placental weight and placental-foetal weight ratio

N = 436	Malaria Absent n = 225		Malaria Present n = 211		P value
**Birth weight (kg)**	3.03	0.4	2.87	0.4	**0.007**

**Birth length (cm)**	48.97	2.4	48.35	2.0	**0.005**

**OFC (cm)**	34.42	1.3	34.07	1.1	**0.028**

**MUAC (cm)**	9.98	0.8	9.80	0.9	**0.036**

**Biceps (cm)**	3.70	0.8	3.55	0.6	**0.032**

**Placental Weight (g)**	557.19	111.5	573.65	119.2	0.186

**Placental-foetal weight ratio**	5.56	1.5	5.20	1.2	**0.019**

**Placental Weight (g)**	557.19	111.5	573.65	119.2	0.186

**Placental-foetal weight ratio**	5.56	1.5	5.20	1.2	**0.019**

Variables expressed as Mean (SD)

OFC, Occipito-frontal Circumference;

MUAC, Mid-upper arm circumference

Cord blood IGF-I levels were significantly lower in babies of mothers with malaria, with no differences in cord blood lipids, insulin and TNF (Table 4).

**Table 4:** Effect of maternal malaria on cord blood biochemical markers

N = 187	Malaria Absent n = 90		Malaria Present n = 97		P value
**Total cholesterol **(mmol/L)	1.65	0.82, 5.53	1.66	0.71, 6.78	0.136

**HDL-C **(mmol/L)	0.66	0.32, 2.16	0.63	0.25, 1.91	0.10

**LDL-C **(mmol/L)	0.70	0.03, 3.19	0.68	0.01, 3.64	0.246

**Triglyceride **(mmol/L)	0.79	0.20, 2.77	0.62	0.26, 3.52	0.30

**Insulin **(mU/L)	2.82	0.1, 59.21	2.61	0.26, 50.95	0.677

**TNF **(ng/L)	2.96	0.01, 97.01	2.23	0.03, 61.58	0.769

**IGF-I **(μg/l)	74.2	24.0, 150.0	61.3	24.0, 145.0	**0.031**

**TNF **(ng/L)	2.96	0.01, 97.01	2.23	0.03, 61.58	0.769

**IGF-I **(μg/l)	74.2	24.0, 150.0	61.3	24.0, 145.0	**0.031**

Variables expressed as Median (IQ Range)

HDL-C, High-density Lipoprotein cholesterol;

LDL-C, Low-density lipoprotein cholesterol;

TNFα, Tumour necrosis factor-alpha;

IGF-I, Insulin-like Growth Factor I

### Associations of maternal and cord biochemical markers and maternal malaria with infant size

Maternal total cholesterol and LDL-C were significantly correlated with birth weight at *p* < 0.001 and insulin at *p* < 0.01 (Table 5), while maternal TG and glucose and cord blood insulin and IGF-I correlated with birth weight at *p* < 0.05 (Table 5).

**Table 5:** Correlations (r values) between maternal and cord biochemical markers and birth indices

Biochemical Markers	Birth Weight (kg)	Birth Length (cm)	OFC (cm)	MUAC (cm)
**Maternal Biochemical Markers (n = 467)**				

**Total cholesterol **(mmol/L)	0.194***	0.108*	0.097	0.204***

**HDL-C **(mmol/L)	0.062	0.024	0.015	0.141**

**LDL-C **(mmol/L)	0.179**	0.095	0.080	0.174**

**Triglyceride **(mmol/L)	0.119*	0.079	0.096	0.076

**Glucose **(mmol/L)	0.107*	0.098	0.159**	0.090

**Insulin **(mU/L)	0.150*	0.062	0.023	0.052

**Cord Biochemical Markers (n = 187)**				

**Cord Insulin **(mU/L)	0.181*	0.003	0.085	0.041

**Cord IGFI **(μg/L)	0.169*	0.093	0.054	0.193*

* *p *< 0.05, **p < 0.01, ***p < 0.001

OFC, Occipito-frontal Circumference;

MUAC, Mid-upper arm circumference;

HDL-C, High-density lipoprotein-cholesterol;

LDL-C, Low-density lipoprotein-cholesterol

To determine the overall independent effects on birth weight, three stepwise regression models were derived (Table 6):

**Table 6:** Determinants of birth weight in stepwise regression

Birth weight	ß	P-value
**Model 1** **N = 396**		

Total Cholesterol (mmol/L)	0.172	**0.001**

**Model 2** **N = 187**		

Maternal malaria (No/Yes)	-0.221	**0.007**

**Model 3** **N = 187**		

Maternal malaria (No/Yes)	-0.155	**0.05**

Cord IGF-I (μg/L)	0.157	**0.045**

Cord Insulin (mU/L)	0.158	**0.042**

Model 1 - Maternal Markers and Malaria at recruitment

Model 2 - Cord Markers and Malaria during pregnancy and delivery

Model 3 - Maternal and Cord Markers and Malaria during pregnancy and delivery

1 In the first model, the influence of malaria at recruitment and all maternal biochemical markers on birth weight was evaluated. Maternal TC was a powerful, independent determinant of birth weight (*p *= 0.001), such that for every 0.15 mmol/L increase in TC, there was a 100 g increase in birth weight.

2 In the second model, the influence of maternal malaria through pregnancy and at delivery and all cord biochemical markers on birth weight was examined. Maternal malaria was the only significant determinant of birth weight (*p *= 0.007).

3 In the final model, maternal malaria, and maternal and cord biochemical markers that had shown significant correlations to birth weight were included. Maternal malaria with cord blood insulin and IGF-I were significantly related to birth weight.

## Discussion

### Effect of maternal malaria on maternal and cord blood biochemical markers

In agreement with other studies in various populations, including Nigerian pregnant women, this study showed an overall elevation in serum lipids, except HDL-C, during pregnancy. The elevation was highest in the third trimester [25-27]. Jimenez *et al *reported no significant changes in HDL-C during pregnancy while others report an elevation [28,29]. These increases in TC and LDL-C and low to normal HDL-C are believed not to be atherogenic [25,27] and are likely to be related to energy transfer to the foetus. The cord lipid profiles are similar to those in Manchester children from the hyperglycaemia and adverse pregnancy outcomes (HAPO) study [30] and to those in Ibadan about 30 years ago, but slightly lower than those reported in eastern Nigeria [17,31,32]. Cord HDL-C and TG levels are about half that of adults while TC and LDL-C are about one-third, hence HDL is the major lipid moiety [33,34].

In these pregnant women, there is a marked cholesterol-lowering impact of malaria, including HDL and LDL, but triglyceride levels were unaffected. These findings corroborate older reports of lower TC and HDL associated with acute malaria but in that setting higher TG [35]. A study of lipid profiles associated with acute malaria in non-pregnant people has shown higher TG but no change in TC [36]. The mechanisms involved in changes in lipid profile associated with malaria are still unclear. *In vitro *experiments have shown a selective uptake of HDL-C by *Plasmodium falciparum *indicating that the HDL-C fraction appears to be a major lipid source for parasite growth [37] and increased intra-parasitic cholesterol and phospholipid levels [38]. *P. falciparum *is unable to synthesize lipids required during its erythrocytic cycle. Hence there is lipid transport through membrane flux into the parasite. Therefore, malaria in pregnancy results in demands for cholesterol from three sources including: the parasite for its growth and probably its attachment properties; the placenta for production of progesterone to maintain the pregnancy, and the fetus itself for growth [37,38]. This combination may lead to low cholesterol levels resulting in prematurity and low birth weight babies.

Maternal plasma glucose and insulin were unaltered by malaria; the result contrasts with a previous study in pregnant women with acute malaria, who had hypoglycaemia associated with increased glucose turnover, attributed to enhanced pancreatic β-cell function [39]. Maternal TNF concentrations in the second trimester were nine-fold higher in those with malaria, but only doubled if malaria occurred in the third trimester. This higher TNF was found previously in Malawian and Kenyan pregnant women in peripheral and placental blood [21,40]. However no associations between TNF and any of the other biochemical parameters or birth size were found.

In contrast this study indicated that malaria had a less pronounced effect on cord blood parameters: cord lipid profile, insulin and TNF levels were not altered, findings not previously described in an endemic malaria area. However cord IGF-I levels were lower.

### Effect of maternal malarial on birth indices

In this study, malaria in pregnancy was associated with younger age, first pregnancy and maternal anaemia, as well as smaller, shorter, thinner babies with smaller head circumferences as previously reported [41,42].

There was no significant difference between the placental weight in infants of mothers with malaria and those without as previously reported by Mcgregor *et al *[43]. The finding of lower placenta-foetal weight ratio in infants born to women with malaria parasitaemia confirms previous reports [44,45]. Placental efficiency can be assessed using the placenta-foetal weight ratio and the lower ratio in babies of mothers with malaria suggests that their placentas have impaired function.

In babies of mothers with malaria, mean birth weight was lower by 5.3% compared to babies of mothers without malaria, biceps skinfold thickness by 4.1% and MUAC by 1.8%, while birth length and OFC were lower by only 1.3% and by 1% respectively (Table 3). This agrees with various studies on the impact of malaria in pregnancy on birth weight (more so than birth length) which showed that mean birth weights of babies born to mothers with malaria were lower by 105 g [46], 371 g [47], 382 g [48] and 461 g [49]. This reduced birth weight is probably due to placental insufficiency resulting from malaria, which leads to intrauterine growth restriction [45]. There are reports of higher positive rates of malaria parasite and/or malaria pigment in the placentae of LBW babies [45,50,51]. In pregnant women with malaria, it has been observed that there is accumulation of infected red blood cells and increased maternal phagocytic cells, especially monocytes in the maternal vascular area of the placenta (the intervillous space) to much higher densities than in the peripheral blood. In addition, there is placental sequestration of the trophozoite and schizont stages, which are absent from the circulation [52]^. ^The inadequate uteroplacental and umbilical blood flow, and alterations in the transplacental transfer of glucose or production of foetal insulin result in LBW babies due to foetal growth restriction [53]. It has been suggested that disturbed placental folate metabolism, placental lactogen, IGF/somatomedin, and somatostatin-like substances, which influence foetal growth, may also play an important role [54].

Very few studies have assessed the impact of malaria in pregnancy on other growth parameters at birth apart from birth weight. In agreement with our findings, Kalanda *et al *documented significantly lower mean birth weight, length, head circumference and mid upper arm circumference in babies of mothers with malaria [53].

### Determinants of birth size

Birth weight remains the single most important determinant of neonatal and infant survival and health. It is therefore important to understand factors that may influence birth weight. In this study, birth weight was strongly related to maternal total cholesterol, LDL-C, and less so to maternal triglyceride, glucose and insulin. This is in contrast to findings in Nigerian newborns about 15 years ago when maternal lipid samples were obtained at delivery but not during pregnancy [31,33]. This study now indicates that variations in maternal cholesterol metabolism appear to have a significant effect on foetal growth during pregnancy and hence birth size. Maternal TG has a lesser impact, although in women with altered glucose tolerance, serum TG concentrations have been shown to predict birth weight [11,13]. Additionally maternal glucose and insulin have a less important impact on foetal growth. In cord blood, insulin and IGF-I were related to birth weight. Both insulin and IGF-I are major growth factors in foetal life as shown by monogenic disorders that affect foetal insulin secretion [55] and IGF-I generation [56-58].

The regression models highlight the most significant factors that determine birth weight, including maternal total cholesterol, malaria status and cord insulin and IGF-I levels. These findings are corroborated by a recent report that showed that maternal and cord IGF-I were reduced in women with placental malaria compared to those without [59].

## Conclusions

In the setting of endemic malaria, maternal total cholesterol during pregnancy and cord blood insulin and IGF-I levels are important markers of foetal growth restriction and reduced birth size likely mediated through placental insufficiency.

## Competing interests

The authors declare that they have no competing interests.

## Authors' contributions

OA participated in overall study conception and design, data collection, analysis, interpretation and manuscript preparation. AW was involved in laboratory assay and data analysis. WB was involved in data collection. OJ participated in data collection. JC and PC both participated in overall study conception and design, data interpretation and manuscript preparation. All authors read and approved the final manuscript.
